# Chemicals for Household Use

**Published:** 1890-09

**Authors:** 


### CHEMICALS FOR HOUSEHOLD USE.

It is surprising, considering how many women have been instructed in chemistry in their school days, to find how few housekeepers make any use of chemicals in various household processes. Especially is this the case in cleansing processes. The washing of clothes is usually wholly accomplished by rubbing the clothes on the washboard, and with no other detergent than soap. The rubbing of the clothes wears them out far more than use, and if housekeepers only knew, or if knowing they would take advantage of the fact that many washing compounds will almost entirely cleanse clothes which are soaked in them over night, and thus almost entirely do away with the labor and wear of the wash-board, wash day might be robbed of half its terrors. Receipts for washing fluids, the principal ingredients of which are soda-ash, ammonia and lime, can be found in nearly every household receipt book and are very cheap and harmless. All such washing compounds are useful and convenient for cleaning woodwork, paints and carpets in a house ; also in washing dishes and securing that desideratum of housekeepers, clean dish cloths. Ammonia is a simple, cheap and harmless chemical that should be bought by the quart and kept in every family. A few drops added to water will cleanse children's hair and make it soft and sweet; it is an admirable disinfectant to remove the odor of perspiration; it will remove grease spots from clothing and often restore colors to stains. Its common and frequent use cannot be too frequently urged.

Borax is another chemical that should find a common use in every family. For cleansing the teeth and sweetening the breath a few grains of the powder in water are unexcelled. It also softens and whitens flannels.

Salicylic acid is a perfectly odorless and harmless yet powerful disinfectant, and for many disinfecting uses in the household is valuable. It is very cheap and convenient in form. In these days, when to stay various forms of disease is so important, mothers and housekeepers would do well to give study and thought to these things, and to try to make their knowledge of science practical. It is very encouraging to note how many women are at present turning their attention to studies in general and applied science. Let us have its benefits exemplified in the household.

